# Supplementary material for: Socioeconomic inequalities in high-risk fertility behaviour among Nigerian women: A non-experimental population-based study
Source: PLoS One. 2024 Nov 18;19(11):e0312028. doi: 10.1371/journal.pone.0312028 (PMC11573142; doi:10.1371/journal.pone.0312028)
Supplement: Attachment — Submitted filename: Point-by-Point Response.docx [file pone.0312028.s001.docx]

September 12, 2024

**Manuscript ID**: PONE-D-24-23186R1

**Title:** **“**Socioeconomic inequalities in high-risk fertility behaviour among Nigerian women: a non-experimental population-based study**”**

RE: Revised manuscript submission and response to reviewers’ comments

Dear Editor,

This letter is in reference to your email dated September 12, 2024 with reviewers’ comments. I am very pleased that the manuscript is potentially acceptable for publication in PLOS ONE once the revisions have been carried out.

I would like to thank the reviewers for these insightful and helpful comments and for giving me the chance to revise our manuscript. I believe the revised manuscript has been significantly improved and the reviewers’ comments have been addressed adequately. I think in its current form it will make a valuable contribution to the literature on this increasingly important topic. 

Please find for your kind consideration the followings:

1) A section-by-section response to the comments and suggestions of the reviewers (below).
2) The revised manuscript, provided as a marked-up copy and/or a clean copy.

I hope that these changes meet with your favourable consideration. Please do not hesitate to get in touch if you require any further information. 

Michael Ekholuenetale

Corresponding Author 

**Reviewers' comments:**

Reviewer #1: 1. There seems to be over-usage of the word "poor" throughout the manuscript, especially with respect to healthcare systems. To many, this might be condescending, albeit being true. Instead use 'low-resource/resource constraints or similar connotations.

**Response**: Thank you very much for the insightful comment. This has been corrected throughout the manuscript.

2. Inconsistent use of verb tense throughout the manuscript. Since the data is cross-sectional, and has already been collected, and the analysis has already been done, use past tense preferably.

**Response**: The verb form has now been corrected throughout the manuscript. Thank you very much for the insightful comment.

3. The Introduction, Results and Discussion, and in fact the whole study while statistically sound, does not explicitly acknowledge that High Risk Fertility Behaviour could be due women's bargaining power in the household as well as the society. While the author(s) briefly mention gender and patriarchial norms in Nigerian society, it will be helpful, if it is mentioned in the limitations, that the role of partners in HRFB was outside the scope of this study, as partners might also have a pivotal influence on HRFB.

**Response**: As recommended, the limitations to the study has been revised to indicate that the role of partners in HRFB was outside the scope of this study. Thank you very much for the insightful comment.

4. The fluidity of writing could be improved, in the introduction and discussion, with incorporation of some theoretical basis to the risk factors selected for analysis.

**Response**: Thank you very much for the insightful comment. The theoretical basis for the selection of risk factors of HRFB has now been included in the methods section.

Reviewer #2: The manuscript presents an important finding of social importance in Nigeria. The author has analysed the dataset properly and the manuscript is written in an intelligible fashion. Although a secondary data analysis, the statistical robustness of analysing and the issue highlighted in the article makes it suitable for publication.

**Response**: Thank you very much for the insightful comment.

Reviewer #3: The study is nicely conceptualized and contributes to reducing HRFB of Nigerian women. I request to author to clarify on following comment:
1. What advantages does decomposition analysis have over multivariate regression analysis in examining socioeconomic inequalities in this study?

**Response**: To clarify the advantages of decomposition analysis over multivariate regression analysis in examining socioeconomic inequalities as in this study;

Decomposition analysis and multivariate regression are both useful in examining socioeconomic inequalities, but decomposition analysis offers several distinct advantages in this study. For example, a) breakdown of inequality: decomposition analysis provides a clearer breakdown of the contribution of education and wealth to overall inequality. This allows for a more detailed understanding of how each variable contributes to socioeconomic inequalities, something multivariate regression cannot easily offer; b) quantifying contribution: decomposition analysis quantifies how much of the inequality in high-risk fertility behaviour (HRFB) is attributable to differences in the distribution of socioeconomic factors versus differences in their effects. This provides insight into whether inequalities are driven more by structural factors or by unequal treatment or outcomes; c) focus on inequality: while multivariate regression primarily examines relationships between variables and an outcome, decomposition analysis specifically focuses on understanding inequality. It is better suited for examining the distribution of an outcome across different groups; d) explaining gaps: decomposition analysis is particularly useful for explaining gaps between groups by isolating how much of the gap is due to differences in characteristics; e) policy-relevant insights: by identifying the specific drivers of inequality, decomposition analysis provides policymakers with clearer insights into which factors to target for interventions to reduce inequalities. Multivariate regression might indicate relationships, but it does not as clearly prioritize interventions based on the contributions of different variables.

In contrast, multivariate regression is more general in assessing associations and interactions among multiple variables but does not provide the same level of specificity in isolating the drivers of inequality. Thank you very much.
